# Ultrasonic-assisted *in situ* synthesis of MOF-199 on the surface of carboxylated cellulose fibers for efficient adsorption of methylene blue†

**DOI:** 10.1039/d4ra02099e

**Published:** 2024-05-08

**Authors:** Zhanpeng Liang, Yuehui Liang, Pengjun Yu, Xin Wang

**Affiliations:** a Inner Mongolia Agricultural University College of Material Science and Art Design Hohhot Inner Mongolia China wangxin2931@163.com; b Inner Mongolia Key Laboratory of Sandy Shrubs Fibrosis and Energy Development and Utilization Hohhot Inner Mongolia China

## Abstract

A high-efficiency porous adsorbent, MOF-199/carboxylated cellulose fibers (MOF-199/CCF), was synthesized *in situ* at room temperature through carboxylation modification, simple sonication, and vacuum drying. The sonication method produced small MOF-199 particles (tens of nanometers), which allowed for uniform distribution of MOF-199 on CCF and improved its efficiency. The presence of CCF carriers reduces the agglomeration of MOF-199 and enhances its performance. The BET-specific surface area of MOF-199/CCF is 264.83 m^2^ g^−1^, which is much larger than that of CCF (2.31 m^2^ g^−1^), proving the successful modification of CCF by MOF-199. MOF-199/CCF exhibits better adsorption capacity than CCF, with an adsorption capacity of 659.6 mg g^−1^ of methylene blue within 30 minutes, and good recycling performance. This work presents a straightforward method for preparing efficient cellulose-based adsorbent materials and offers a novel approach for synthesizing MOF composites.

## Abstract 2

Efficient methylene blue sorbents achieved through simple, rapid, and safe sonication.

## Graphical abstract

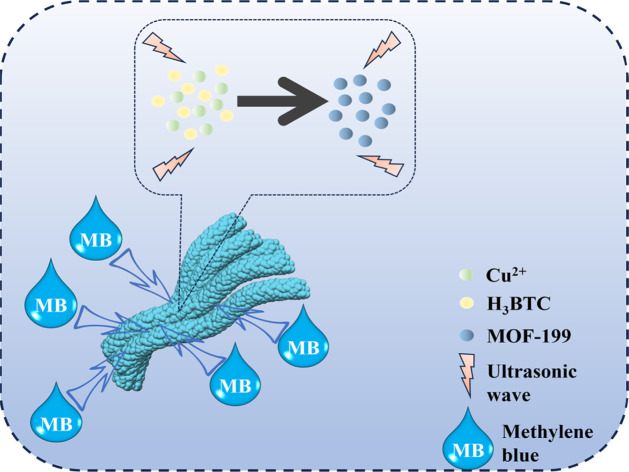

## 1 Introduction

The issue of water pollution is becoming increasingly severe with the acceleration of industrialization worldwide.^1^ According to the United Nations, by 2025, at least 1 billion people living in water-scarce areas will face a water crisis. Industrialization has introduced many emerging water pollutants, including heavy metals, organic pollutants, drug molecules, and radionuclides. These pollutants have a significant negative impact on the environment and are now a serious global concern. Organic dyes are a prevalent class of pollutants that have significant impacts. They are a typical type of pollutant.^2^ Approximately 7 × 10^5^ tons of commercial organic dyes are produced worldwide each year, with approximately 2% being discharged into various bodies of water. These dyes, particularly aromatic molecules, are biologically non-degradable and possess mutagenic and carcinogenic properties. Even at low concentrations, they can have harmful effects on the environment, aquatic ecosystems, and human health.^3^ Methylene blue is a common type of organic dye and it is particularly problematic due to its complex structure and chemical properties, which make it difficult to degrade and cause significant harm to the water environment.^4^ To effectively address water pollution and the water crisis, various methods have been employed, such as chemical precipitation, membrane separation, adsorption, ion exchange, deep oxidation, biodegradation, flocculation, desalination and photocatalytic degradation.^2,5^ Adsorption methods are commonly used in wastewater dye treatment due to their low cost, simple conditions, and minimal secondary pollution. However, many adsorbents have significant drawbacks, such as high cost, complex preparation processes, low adsorption capacity, and poor biocompatibility, which greatly hinder their practical application.^6,7^ The development of a simple, efficient, and inexpensive adsorbent is an effective solution to the existing problems.

Cellulose, the most abundant polysaccharide in nature, is often used in the field of water pollution control due to its large aspect ratio, abundant functional groups, excellent mechanical properties, water stability, and incomparable environmental friendliness.^8^ The adsorption capacity of cellulose materials is influenced by their size. Smaller cellulose materials generally have stronger adsorption capacity. However, smaller size often indicates complex and harsh preparation conditions, which are not suitable for large-scale applications.^9^ Therefore, it is more time-saving and economical to enhance the adsorption performance of larger cellulose materials.

Carboxylated cellulose fibers (CCF) are cellulose fibers that have been selectively oxidized at the C6 position to form carboxyl groups through an oxidation treatment.^10^ This modification results in a surface with abundant carboxyl functional groups and a preparation process that is more time-efficient than that of nanocellulose, making it a more economical option. The larger size of the CCF is convenient for loading other materials to prevent them from falling off due to their small size. Additionally, the functional groups on the surface of the CCF, such as carboxyl and hydroxyl groups, can form chemical bonds with other materials through chemical action, achieving a firm load and improving the CCF.

Metal–organic frameworks (MOFs) are a type of three-dimensional porous organic–inorganic hybrid material. They have abundant porosity and an extremely high specific surface area. The structure and size of MOFs can be adjusted by changing the introduced organic ligands and different reaction conditions.^11^ MOFs are widely used in the field of water pollution control due to these advantages. MOF-199, also known as HKUST-1, is a type of MOF that exhibits exceptional adsorption capacity for cationic dye MB.^12^ Therefore, MOF-199 is frequently utilized to address water pollution caused by MB. In most studies, researchers typically use the solvothermal method to prepare MOF-199 materials. This method requires the reactor to react continuously at a high temperature (>100 °C) for dozens of hours, which is time-consuming, labor-intensive and has potential safety hazards. Researchers also use the simple and safe method of stirring at room temperature to synthesize MOF-199. However, this method is time-consuming, taking more than 20 hours.^13^ To address these issues, we utilized the ultrasonic method at room temperature to prepare MOF-199 materials. Small-scale, small-size MOF-199 can be successfully prepared in just 30 minutes using ultrasonication, this method significantly reduces preparation time. Meanwhile, ultrasonication-prepared MOF-199 overcomes size limitations due to its small size, expanding its range of potential applications. However, its irregular shape and small size make it prone to agglomeration, which significantly impacts its performance.^14^ To address the issue of MOF-199 agglomeration and enhance the adsorption capabilities of cellulose materials, we achieved the *in situ* synthesis of MOF-199 on CCF through ultrasonication. This gentle, efficient, and safe process resulted in the creation of an MB adsorbent with high adsorption capacity.

Our approach deviated from the traditional solvothermal method and instead utilized the ultrasonic method, which has received little attention in this field. The surface carboxyl content of micron-sized large-size cellulose fibers was increased through selective oxidation. Subsequently, the carboxyl groups were chemically bonded to Cu^2+^ (MOF precursor), and organic ligands were introduced to achieve *in situ* synthesis of MOFs on CCF under ultrasonic action. Simultaneously, the introduction of CCF can provide a dispersed carrier for small-sized MOF-199, significantly reducing its agglomeration and achieving a synergistic effect between the two. The MOF-199/CCF composite exhibits high adsorption capacity and excellent recyclability for MB. This study aims to offer technical and theoretical support for designing and fabricating cellulose-based adsorbent materials that are simple, time-saving, safe, and have high adsorption capacity. Additionally, it explores the field of ultrasonic preparation of MOF composites.

## 2 Experimental

### 2.1 Materials

The cellulose fibers are obtained from Salix timber grown in the Inner Mongolia Autonomous Region of China and processed in the laboratory. 2,2,6,6-Tetramethylpiperidin-1-oxyl radical (TEMPO, 98%), sodium bromide (NaBr, 99%), sodium hypochlorite solution (NaClO, 6–14% active chlorine basis), anhydrous copper acetate (Cu(CH_3_COO)_2_, 98%), trimesic acid (H_3_BTC, 98%) and methylene blue (MB, 98%) are all from Macklin Company (Shanghai, China). Sodium hydroxide (NaOH, 99%), sodium carbonate (Na_2_CO_3_, 99%), sodium bicarbonate (NaHCO_3_, 99%), hydrochloric acid (HCl, 36–38%) are all from Tianjin Fengchuan Chemical Reagent Technology Co., Ltd (Tianjin, China). All other solvents were not further purified.

### 2.2 Preparation of MOF-199/CCF

#### 2.2.1 Preparation of CCF

Based on previous research, the method was improved.^15,16^ To prepare the cellulose suspension, dissolve 7 g of Na_2_CO_3_ and 3 g of NaHCO_3_ in 500 ml of deionized water. Then, add 0.1 g of TEMPO and 0.5 g of NaBr, stirring until completely dissolved. Finally, add 5 g of cellulose fibers to the solution. To adjust the pH of the suspension to approximately 11, NaClO solution and hydrochloric acid were utilized. The reaction was stirred at 35 °C for 6 hours, and 10 ml of absolute ethanol was added to stop the reaction. The cellulose fibers were then washed with deionized water until neutral to obtain CCF.

#### 2.2.2 Preparation of MOF-199/CCF using ultrasonic techniques

The precursor A of MOF-199/CCF was obtained by dissolving 1 mmol of Cu(CH_3_COO)_2_ in 20 ml of deionized water, adding 0.2 g of CCF to the solution, sonicating at a power of 300 W for 10 min, and then stirring at a speed of 200 r min^−1^ for 20 min. A suspension was obtained by dissolving 1 mmol of H_3_BTC in 20 ml of absolute ethanol (precursor B), mixing it with precursor A, stirring the solution at a speed of 200 r min^−1^ for 5 minutes to ensure even mixing, and then subjecting the mixed solution to ultrasonic cleaning at a power of 100 W for 30 minutes. The suspension should be centrifuged at a speed of 4000 r min^−1^ for 10 minutes. After pouring off the upper liquid, deionized water should be added to the precipitate and shaken evenly. The same centrifugation process should be repeated 5 times. Then, the deionized water should be replaced with absolute ethanol and washed 5 times to remove any residual Cu^2+^ and H_3_BTC. The centrifuged precipitate was then placed in a vacuum-drying oven and dried at 60 °C for 12 hours. After complete drying, it was ground with a mortar and sieved with a 100-mesh sieve to obtain MOF-199/CCF. Pure MOF-199 was prepared using the same process but without the addition of CCF.

### 2.3 Characterization

In this study, we used a Bruker INVENIO S FT/IR spectrophotometer to measure the IR spectrum with a wavelength range of 4000–400 cm^−1^. Additionally, we employed a Thermo Scientific K-Alpha spectrometer to measure X-ray photoelectron spectroscopy. X-ray diffraction (XRD) spectroscopy was measured using an XRD-6000 (Shimadzu, Japan) diffractometer with a measurement range of 5°–45°. Thermogravimetric (TG) analyses of the samples were manipulated using a TG 209 F1 Libra (NETZSCH, German) analyzer heated from room temperature to 800 °C under atmosphere at the heating rate of 20 °C min^−1^. Nitrogen adsorption assays were conducted using a specific surface pore size analyzer (SSA-4200) after 12 hours of vacuum degassing at 313 K. The morphology of the material was observed using scanning electron microscopy (SEM, Phenom Pro). The nanoparticle size potentiometer BeNano 90 Zeta was used to measure both the zeta potential and particle size distribution. Cu elemental content was determined using an inductively coupled plasma emission spectrometry detector (ICP, 5110VDV).

### 2.4 Adsorption experiments

The construction of a calibration curve for the MB standard solution may be achieved through the utilisation of a UV-Vis spectrophotometer. This instrument is employed to measure the absorbance of the standard solution at a wavelength of 664 nm. The linear relationship between the absorbance of the solution and its concentration is then established. The absorbance of the adsorbed solution is subsequently measured to determine the concentration of the solution after it has been diluted proportionally. All adsorption experiments were conducted using 20 mg of sorbent in 100 ml of solution. The total adsorption time was set to 30 minutes, and the oscillation rate was set to 160 r min^−1^. The pH of the MB solution was adjusted using 0.1 M NaOH and 0.1 M HCl solutions. All adsorption experiments were conducted at 298 K and pH 7, with an initial MB concentration of 100 mg L^−1^, unless otherwise specified. After adsorbing MB, the adsorbent was ultrasonicated in anhydrous ethanol and the process was repeated until the supernatant became colourless. Then, it was rinsed with deionised water and placed in a vacuum oven at 60 °C for drying. The dried adsorbent was used for the next adsorption cycle. The adsorption capacity of the adsorbent for MB was calculated using eqn (1):^17^

1 *q*_*t*_ = *V* (*c*_0_ − *c*_*t*_)/*m*

where *V* (L) is the volume of the MB solution; *c*_0_ (mg L^−1^) and *c*_*t*_ (mg L^−1^) are the initial and residual liquid concentrations at time *t* (min), respectively; and *m* (g) is the mass of the adsorbent. The adsorption capacity at adsorption equilibrium is defined as the saturated adsorption capacity (*q*_e_, mg g^−1^).^12^

The pseudo-first-order and pseudo-second-order kinetic models are employed to fit the adsorption kinetics of MOF-199/CCF toward MB, shown as eqn (2) and (3):^18^

2 ln(*q*_e_ − *q*_*t*_) = ln *q*_e_ − *k*_1_ × *t*

3 *t*/*q*_*t*_ = 1/*k*_2_ × *q*_e_^2^ + *t*/*q*_e_

where *q*_e_ (mg g^−1^) and *q*_*t*_ (mg g^−1^) are the adsorption capacities at equilibrium and time *t* (min); and *k*_1_ (min^−1^) and *k*_2_ (g mg^−1^ min^−1^) are the pseudo-first-order and pseudo-second-order rate constants, respectively.

In this study, we present an intragranular diffusion model for the adsorption of MB onto MOF-199/CCF, as described in eqn (4):^19^

4 *q*_*t*_ = *k*_i_ × *t*^1/2^ + *C*

The diffusion rate constant within the particle is represented by *k*_i_ (mg g^−1^ min^−1/2^), and the intercept, which represents the boundary layer resistance, is represented by *C*. When the adsorption process involves diffusion within particles, the relationship between *q*_*t*_ (mg g^−1^) and *t*^1/2^ (min^1/2^) is linear, and *k*_i_ and *C* can be obtained from the linear fitting curve.

The Langmuir and Freundlich adsorption Isotherm models were used to analyze the adsorption of MB on MOF-199/CCF, shown as eqn (5) and (6):^20^

5 *c*_e_/*q*_e_ = *c*_e_/*q*_m_ + 1/*q*_m_ × *k*_L_

6 ln *q*_e_ = ln *k*_F_ + 1/*n* × ln *c*_e_

where *k*_L_ (L mg^−1^) and *q*_m_ (mg g^−1^) are the Langmuir constants related to *q*_e_ (mg g^−1^) for a complete monolayer and energy of adsorption, respectively. *K*_F_ (mg g^−1^) and *n* are the Freundlich constants that indicate the adsorption capacity and intensity, respectively. The residual concentration of MB at adsorption equilibrium is represented by *c*_e_ (mg L^−1^).^20^

The thermodynamic parameters of MB adsorption by MOF-199/CCF at different temperatures can be calculated by eqn (7) and (8):^20^

7 ln(*q*_e_/*c*_e_) = Δ*S*/*R* − Δ*H*/*R* × *T*

8 Δ*G* = Δ*H* − *T*Δ*S*

where Δ*H* (kJ mol^−1^) and Δ*S* (J mol^−1^ K^−1^) are the enthalpy change and entropy change during the adsorption process, and Δ*G* (kJ mol^−1^) is the Gibbs free energy change. *R* (8.314 J mol^−1^ K^−1^) is the gas constant. *T* (K) is the absolute temperature.

## 3 Results and discussion

### 3.1 Synthesis process of MOF-199/CCF

**Fig. 1:**
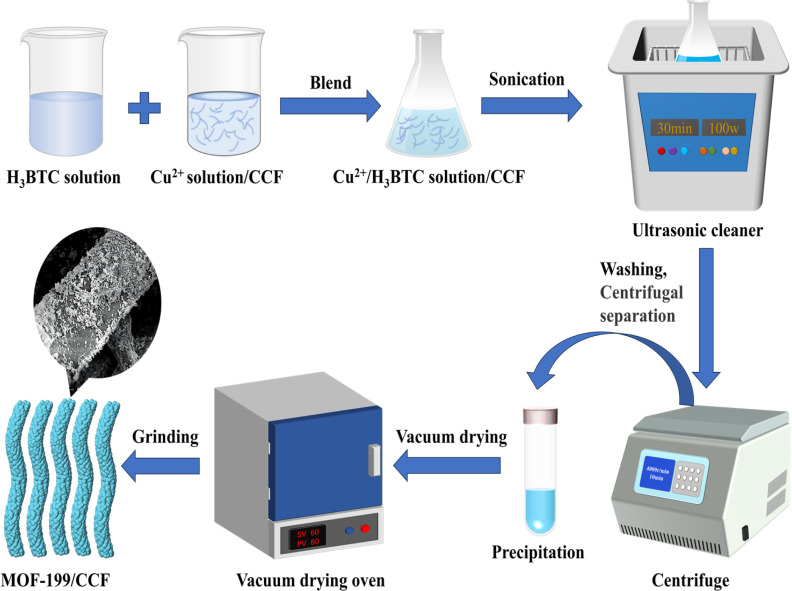
Preparation process of MOF-199/CCF.

Fig. 1 shows the synthesis route of MOF-199/CCF. The synthesis of MOF-199/CCF involved the selective oxidation of cellulose fibers using the TEMPO/NaClO/NaBr oxidation system. First, NaClO oxidized the TEMPO radicals to nitrosas ions. Then, the primary hydroxyl group on the cellulose macromolecular chain attacked it nucleophilically to form hydroxylamine. Finally, the hydroxyl group on cellulose C6 was oxidized to a carboxyl group,^10^ resulting in the preparation of CCF. The carboxyl groups on CCF facilitate the binding of CCF and Cu^2+^ in Cu(CH_3_COO)_2_ solution through electrostatic adsorption and chelation.^21^ The introduction of H_3_BTC and Cu^2+^ on the CCF surface through ultrasonic generates MOF-199 *in situ*. Under the influence of ultrasound, tiny bubbles form in the suspension. These bubbles are sequentially generated, grown, and burst due to hot spots, resulting in extreme local hyperthermia and high pressure.^22^ Due to these conditions, enough energy can be acquired to cause the collapse of particles near the surface of the solid, leading to the fragmentation of larger particles or the deagglomeration of nanoparticles.^14^ This results in the formation of nanoscale MOF-199 and ensures a uniform distribution of MOF-199 on the CCF.

### 3.2 Characterization of MOF-199/CCF

**Fig. 2:**
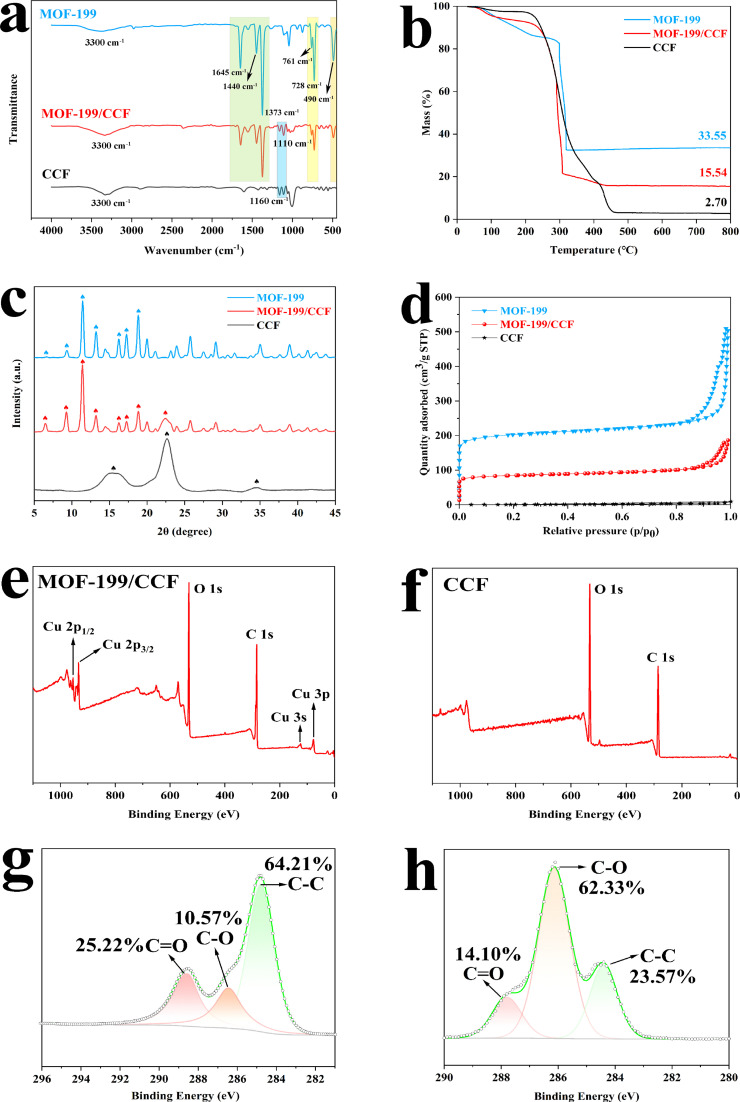
FTIR (a); TG (b); XRD (c); N_2_ adsorption (d); XPS spectra of MOF-199/CCF and CCF (e) and (f); fine XPS scanning spectrum of the C element (g) and (h).

FTIR was utilized to identify the chemical functional groups of the materials and their interactions, as depicted in Fig. 2(a) and S1.† Fig. S1† shows the infrared spectra of CCF *versus* untreated virgin cellulose fibers (CF), CCF shows a distinct infrared characteristic peak at 1600 cm^−1^ compared to CF, which is attributed to the asymmetric contraction vibration of –COO–, indicating successful oxidation of CF.^23^ The peak around 3300 cm^−1^ is attributed to the O–H stretching vibration, this comes from physically adsorbed water.^24^ The bands at 490 cm^−1^ in MOF-199 and MOF-199/CCF are attributed to the Cu–O metal–oxygen bond, and the peaks at 728 cm^−1^ and 761 cm^−1^ are attributed to =C–H stretching. The peaks at 1160 cm^−1^ and 1110 cm^−1^ are generated by the stretching vibrations of the C–C backbone and C–O groups in cellulose molecules, these are the typical infrared spectral characteristic peaks of CCF.^25^ The peaks at 1373 cm^−1^ and 1440 cm^−1^ are attributed to the symmetric and asymmetric vibration of O=C–O,^26^ this is related to H_3_BTC, while the peak at 1645 cm^−1^ is attributed to the C=O stretching vibration, this is also related to H_3_BTC.^27,28^ These peaks are characteristic of MOF-199, and their appearance on MOF-199/CCF confirms the coordination of organic linkers with Cu^2+^ has been successfully achieved.

Thermogravimetric analysis was utilized to calculate the mass loading rate of MOF-199 on CCF. Fig. 2(b) shows the ash content of the three materials as a function of temperature. MOF-199 exhibited the highest ash content (33.55%), while CCF had the lowest ash content (2.70%). The ash content of MOF-199/CCF, prepared by the two composite materials, was in the middle (15.54%). The percentage of MOF-199/CCF components was calculated based on ESI,†,^29^ the mass loading rate of MOF-199 is 41.62 wt%. Concurrently, an ICP test was conducted on MOF-199/CCF, and the test results indicated that the Cu element constituted 12.655% of MOF-199/CCF. The chemical formula of MOF-199 (C_18_H_12_O_15_Cu_3_) indicates that the generation of MOF-199 is 3.43 times greater than that of Cu.^29^ The calculated mass ratio of MOF-199 in MOF-199/CCF is 43.41 wt%, which is in close agreement with the mass ratio (41.62 wt%) calculated from thermogravimetric analysis data. Although the mass ratio of MOF-199 in MOF-199/CCF is less than 50%, MOF-199/CCF exhibits almost the same excellent adsorption capacity as pure MOF-199. This suggests that the existence of the CCF carrier provides good support for MOF-199 and greatly reduces its agglomeration.

Fig. 2(c) displays the XRD pattern of MOF-199/CCF. The characteristic crystalline broad peak of CCF is observed at 2*θ* = 15.7, 22.5, 34.6°, the diffraction peaks of CCF are related to the crystal planes (110), (200), and (004), respectively, which are typical of cellulose type I structures.^30–32^ Additionally, peaks are observed at 2*θ* = 6.8, 9.5, 11.7, 13.5, 16.5, 17.5, and 19°. The main diffraction peaks of MOF-199 are related to the crystal planes (200), (220), (222), (400), (422), (440), and (511).^33^ The other characteristic peaks of MOF-199/CCF are also highly consistent with the characteristic peaks of MOF-199, and the characteristic peaks of CCF (2*θ* = 22.5°) can also be found in MOF-199/CCF. The crystal structure of MOF-199 was introduced to MOF-199/CCF, confirming the presence of MOF-199 on CCF and the resulting improvement.

Fig. 2(d) displays the N_2_ adsorption–desorption curves for the three materials, and the curves were used to analyze the specific surface area and pore size of the materials.^34^ MOF-199 and MOF-199/CCF exhibit typical type I adsorption curves, with high adsorption capacity in the low-pressure region, indicating a large number of micropores in the material. The H4 hysteresis rings in the high-pressure area indicate the presence of mesopores, mainly generated by particle accumulation. Fig. S2(a)† shows the adsorption curve of CCF, which exhibits a typical type III adsorption curve. Fig. S2(b)† displays the pore size distribution of the three materials. The pore size distribution of MOF-199 and MOF-199/CCF indicates that their pore sizes are primarily in the microporous range with a significant number of mesopores. The pore size distribution curves effectively explain the type I adsorption curves of both MOF-199 and MOF-199/CCF. This is the main reason for their similar adsorption behavior. Table S1† shows that CCF has a BET-specific surface area of 2.31 m^2^ g^−1^ and an average pore size of 12.3 nm. The low specific surface area of CCF is due to its fewer micropores/mesopores.^35^ The specific surface area of pure MOF-199 BET is 645.26 m^2^ g^−1^, and the average pore size is 2.19 nm. The relatively low specific surface area of MOF-199, in comparison to that reported in other works, may be attributed to the small size and apparent agglomeration of MOF-199 obtained by ultrasonic preparation in this study. This results in a significant reduction in the specific surface area due to the clustered structure resulting from layer-by-layer stacking. After the introduction of MOF-199, the specific surface area of MOF-199/CCF reaches 264.83 m^2^ g^−1^, which is 115 times that of pure CCF, and the average pore size is reduced to 2.53 nm. The synthesis of MOF-199 on the surface of CCF was successfully demonstrated, with MOF-199 playing a dominant role in the specific surface area contribution for MOF-199/CCF.^20^

XPS was used to characterize the surface elements and elemental chemical states of MOF-199/CCF and CCF. The XPS spectra of MOF-199/CCF for Cu elements are presented in Fig. 2(e), including Cu 2p_1/2_ at 954 eV, Cu 2p_3/2_ at 934 eV, Cu 3s at 124 eV, and Cu 3p at 77 eV.^36^Fig. 2(f) shows the XPS spectra of pure CCF, which contains only C and O. A comparison of the two spectra proves the existence of MOF-199 on the surface of CCF. Fig. 2(g) and (h) show the fine scanning spectrum of the C element for MOF-199/CCF and CCF, respectively. The C=O peak area is larger than the C–O peak, indicating a higher C=O bond content (25.22%) in MOF-199/CCF compared to the C–O bond (10.57%). However, the C–O bond content (62.33%) in CCF is much higher than that of the C=O bond (14.10%). The introduction of H_3_BTC is the reason for this change, the addition of H_3_BTC has resulted in a change in the C=O bond content, providing evidence of successful coordination between H_3_BTC and Cu^2+^.

**Fig. 3:**
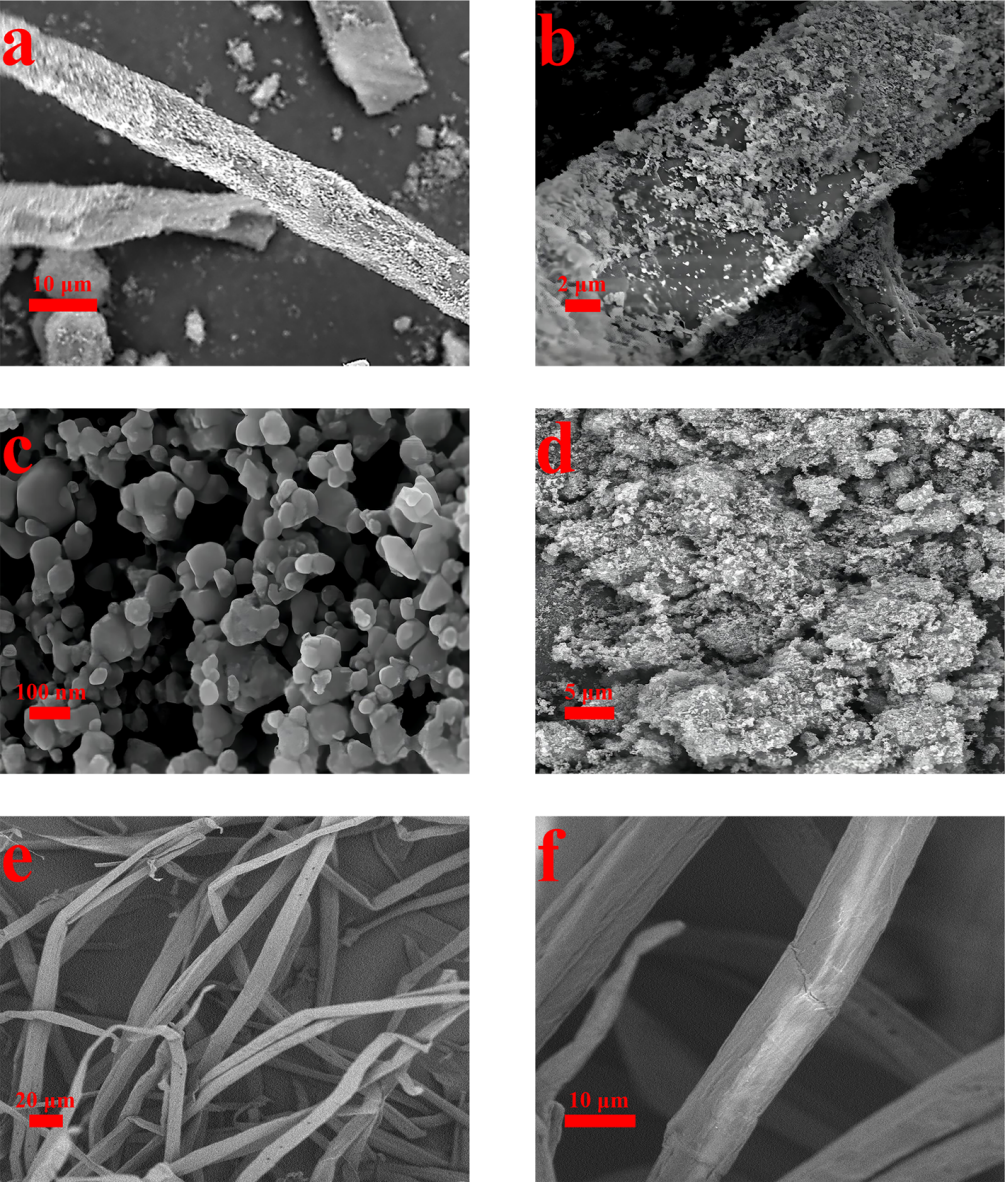
SEM image: MOF-199/CCF (a), (b), and (c); MOF-199 (d); CCF (e) and (f).

Fig. 3 shows the surface topography of the material at different magnifications using SEM. Fig. 3(e) and (f) depict pure CCF. The figures clearly show that the surface of the CCF is both dense and smooth, without any other substances present. As can be seen from Fig. 3(a) and (b), fine MOF particles are distributed on the surface of CCF, and the distribution of MOFs is uniform and dense, this is comparable to Fig. 3(e) and (f), in stark contrast, which proves that it is feasible to prepare MOF-199/CCF composites by ultrasonic method. Fig. 3(c) illustrates that the MOF-199 prepared using the ultrasonic method has a small size, with a diameter of mostly tens of nanometers, which is consistent with the previous report.^14^ In order to obtain detailed size data of MOF-199, the particle size distribution of MOF-199 was measured after sufficient ultrasonic dispersion of MOF-199 in deionised water, Fig. S3† is the particle size distribution graph of MOF-199, and the particle size data showed that the size distribution range of MOF-199 was large, and the main particle sizes were concentrated in the range of 100 nm, which was more consistent with the size distribution of MOF-199 shown in Fig. 3(c). The smaller size is a crucial factor in the even and firm loading of MOF-199 on the CCF surface. The SEM image in Fig. 3(d) shows pure MOF-199 in an agglomerated state, with numerous nanoparticles forming large particle agglomerates that are difficult to disperse in practical use. This greatly impacts its performance. The comparison of Fig. 3(b) and (d) indicates that CCF is an effective dispersion carrier for MOF-199. This reduces the agglomeration of MOF-199 and improves its application performance. Additionally, MOF-199 can be used to modify CCF, further enhancing its performance.

### 3.3 Adsorption properties of MOF-199/CCF for MB

#### 3.3.1 Adsorption properties of various adsorbents for MB

**Fig. 4:**
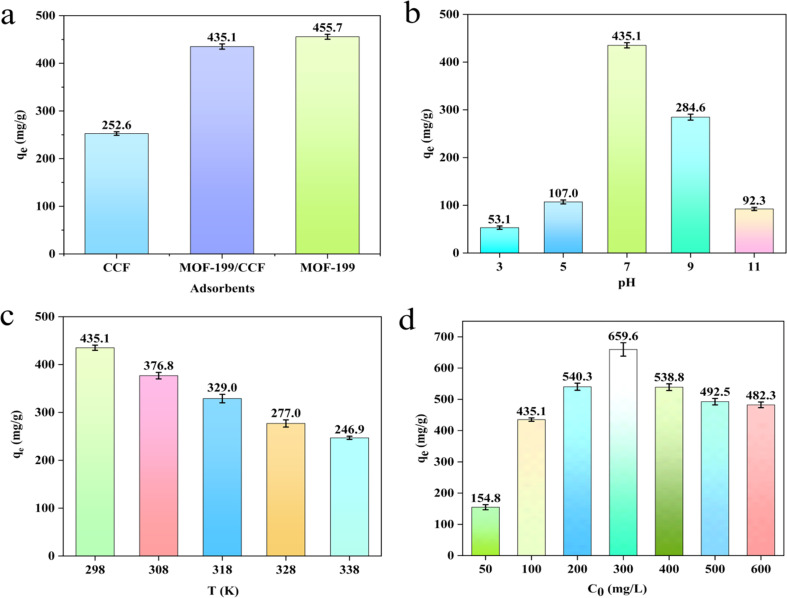
Effects on the adsorption capacity: different adsorbents (a); pH (b); temperature (c); initial concentration (d).

The study investigated the adsorption properties of three different adsorbents for MB, as presented in Fig. 4(a), Fig. S4(a)† illustrates the variation of adsorption with time. Of the three materials, CCF exhibited the lowest adsorption capacity for MB at 252.6 mg g^−1^. This can be attributed to the fact that MB is a cationic dye and CCF has a significant number of carboxyl groups on its surface, which can interact electrostatically with positively charged MB to adsorb it. The adsorption capacity of MOF-199 alone for MB was up to 455.7 mg g^−1^. When MOF-199 was loaded on CCF, the adsorption capacity of MOF-199/CCF increased to 435.1 mg g^−1^, which was close to that of MOF-199. This indicates that the addition of MOF-199 significantly increased the adsorption capacity of CCF. It has been demonstrated that CCF, serving as the carrier of MOF-199, MOF-199 can be effectively supported by it, can significantly enhance its adsorption capabilities while preventing agglomeration. This allows a small amount of MOF-199 to exhibit a high adsorption capacity. In the following experiments, MOF-199/CCF was utilized as the adsorbent.

#### 3.3.2 Effect of pH on adsorption performance

Fig. 4(b) shows the adsorption capacity of MOF-199/CCF for MB under different pH values, and Fig. S4(b)† illustrates the variation of adsorption with time. The adsorption capacity increased initially and then decreased with increasing pH. The maximum adsorption capacity of MOF-199/CCF for MB was 435.1 mg g^−1^ at pH 7. The zeta potential change of MOF-199/CCF is shown in Fig. S5.† MOF-199/CCF has a negatively charged surface due to its isoelectric point (IEP) being at pH = 2.96, while the pH values in the experimental range are all greater than the IEP. This negative charge promotes electrostatic attraction between the positively charged MB and MOF-199/CCF, facilitating adsorption.^37^ The negative charge of MOF-199/CCF increased as the pH value increased. This resulted in a stronger electrostatic attraction between MOF-199/CCF and MB, leading to an increase in adsorption capacity. The adsorption capacity of MOF-199/CCF peaks at pH = 7 and then decreases. This is because the added NaOH, which increases the pH, electrostatically shields and affects the adsorption of MOF-199/CCF, resulting in a reduction of the adsorption capacity.^38^ Therefore, pH = 7 was selected for follow-up experiments.

#### 3.3.3 Effect of temperature on adsorption performance

**Fig. 5:**
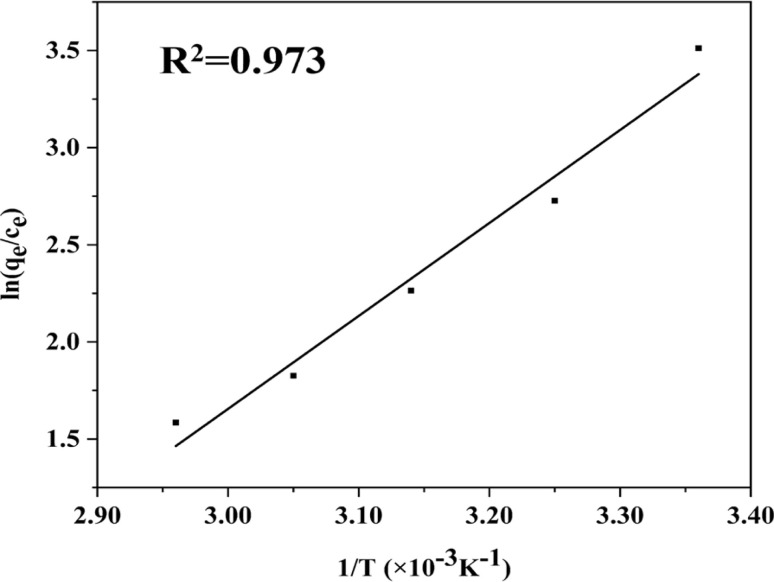
Fitting results of MB on MOF-199/CCF by using thermodynamic model.

Fig. 4(c) displays the adsorption capacity of MOF-199/CCF for MB at various temperatures, and Fig. S4(c)† illustrates the variation of adsorption with time. The adsorption thermodynamic parameters and fitting thermodynamic curves were calculated using eqn (7) and (8), and are presented in Table S2† and Fig. 5, respectively. Table S2† shows that Δ*G* is negative for all temperature conditions and increases with temperature, indicating a spontaneous adsorption process. Δ*H* is also negative, indicating an exothermic adsorption process. Higher temperatures result in a weaker adsorption effect, consistent with experimental results.

#### 3.3.4 Effect of initial concentration of MB on adsorption performance

Adsorption experiments were conducted using various initial concentrations of MB. The adsorption isotherm of MB on MOF-199/CCF was analyzed using Langmuir and Freundlich adsorption models. Fig. 4(d) illustrates the change in the adsorption capacity of MOF-199/CCF at varying initial concentrations of MB, and Fig. S4(d)† illustrates the variation of adsorption with time. The equilibrium adsorption capacity initially increases rapidly with concentration, reaching a maximum of 659.6 mg g^−1^ at an initial concentration of 300 mg L^−1^.

Subsequently, it slowly decreases, with the equilibrium adsorption capacity at 500 mg L^−1^ and 600 mg L^−1^ being almost identical. According to Fig. S4(d),† the adsorbent reached adsorption equilibrium in 15 minutes at an MB concentration of 50 mg L^−1^, while the longest equilibrium time was 24 minutes at a concentration of 100 mg L^−1^. This is because the adsorbent requires a longer adsorption time at higher concentrations. The equilibrium time decreased gradually as the concentration increased. At 500 and 600 mg L^−1^, the equilibrium time decreased to 6 minutes, and the adsorption and desorption phenomena became very intense. As the concentration of MB increases, the high concentration of solute molecules can provide additional impetus for the adsorption of MOF-199/CCF. This promotes the transfer of MB molecules from the solvent to the adsorbent. At high concentrations of MB, the limited adsorption sites on the adsorbent become quickly occupied by the MB molecules due to a strong driving force. This affects the adsorbent and promotes a strong adsorption and desorption process of MOF-199/CCF, resulting in a shorter time required for the adsorbent to reach adsorption equilibrium. As a consequence, the equilibrium adsorption capacity decreases and the concentration of residual MB solution changes significantly after the adsorption equilibrium is reached.

Table 1 and Fig. S6† show the parameters and fitting curves of the Langmuir and Freundlich adsorption isotherm models. The Langmuir adsorption isotherm (*R*^2^ = 0.981) is more suitable for describing the adsorption process of MOF-199/CCF than the Freundlich adsorption isotherm (*R*^2^ = 0.352) due to its higher correlation coefficient and better correlation.^37^ It was determined that the adsorption process of MOF-199/CCF exhibited clear monolayer adsorption characteristics.^6,39^ Only the MB molecules in direct contact with the adsorbent surface were adsorbed, and this adsorption occurred exclusively at a specific site. No other adsorption occurred at the same site, which explains the strong adsorption and desorption at high concentrations.

**Table 1:** Langmuir and Freundlich adsorption isotherm model parameters for the adsorption of MB by MOF-199/CCF

*q*_m_ (mg g^−1^) experimental	Langmuir model			Freundlich model		
	*q*_m_ (mg g^−1^) theoretical	*k*_L_ (L mg^−1^)	*R* ^2^	*n*	*k*_F_ (mg g^−1^)	*R* ^2^
659.6	500	0.87	0.981	5.168	175.53	0.352

#### 3.3.5 Adsorption kinetics

**Fig. 6:**
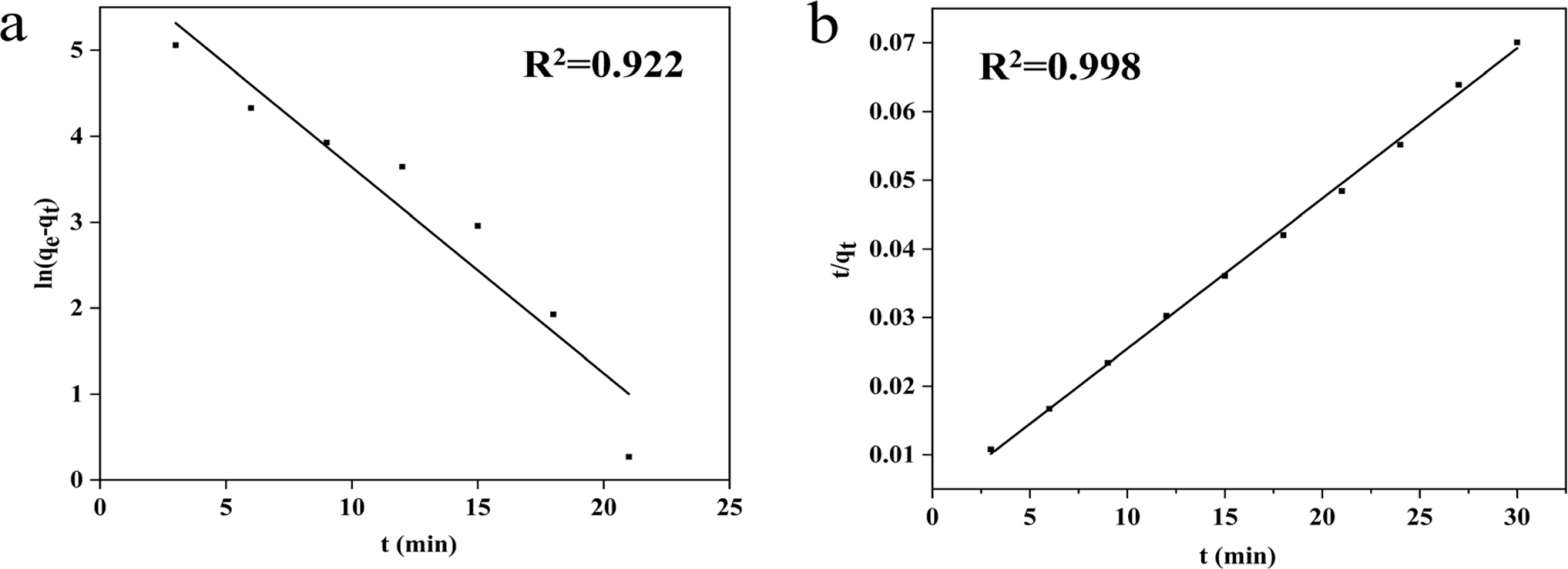
Fitting results of MB on MOF-199/CCF by using pseudo-first-order kinetic (a); fitting results of MB on MOF-199/CCF by using pseudo-second-order kinetic (b).

The study utilized the adsorption kinetics model to examine the adsorption of MB by MOF-199/CCF.^40^Fig. 6 displays the fitting curves of the pseudo-first-order and pseudo-second-order kinetic models. The parameters of these models are presented in Table S3.† The pseudo-second-order kinetic model fitting curve (*R*^2^ > 0.99) agreed well with the experimental data of the adsorption process. This suggests that the adsorption of MB by MOF-199/CCF was dominated by chemisorption, which depended on the electrostatic interaction and weak p–p interaction between MOF-199 and MB molecules.^12,41^ The results of the pseudo-first-order kinetic model were in good agreement with the experimental data (*R*^2^ = 0.922), indicating that the adsorption of MB by MOF-199/CCF was existing physisorption.^20^ This was attributed to the mesoporous structure of MOF-199/CCF, suggesting that the adsorption process of MB by MOF-199/CCF involved a synergistic effect of chemical and physical adsorption.

The study introduced the intra-particle diffusion model to analyze the adsorption mechanism of MOF-199/CCF on MB. Table 2 and Fig. S7† show the parameters and fitting curves of the model. Fig. S7† displays three linear parts with varying linear relationships. The slope of the fitting curve in the first part is the largest, indicating that the adsorption process is the fastest. This is due to the low resistance of the MB molecule to diffuse from the solution to the surface of MOF-199/CCF. The slope of the fitting curve in the second part is smaller than that in the first part, indicating that diffusion occurs within the particles. The intramolecular diffusion of the MB molecule into the pores of the adsorbent becomes larger, resulting in increased resistance and rate limiting. The fitting curve does not pass through the origin, indicating that intra-particle diffusion is not the only cause of rate control. The slope of the fitted curve in the third stage decreases significantly, indicating a sharp decrease in the rate of intraparticle diffusion, reaching the adsorption–desorption equilibrium stage. Table 2 shows the intercepts of the fitted curves for the three phases (*C*_1_ = 483.84, *C*_2_ = 287.57, *C*_3_ = 129.75). These intercepts represent the boundary layer effect at different phases, which is closely related to the size of the adsorption rate. The larger the value of *C*, the more pronounced the boundary layer effect, and the smaller the intraparticle diffusion rate constant. The boundary layer effect becomes more pronounced as the *C* value increases, while the intraparticle diffusion rate constant and adsorption rate decrease. This coincides with the observed change in absorption rate during adsorption.

**Table 2:** Intra-particle diffusion model parameters of MOF-199/CCF adsorption of MB

*k*_1_ (mg g^−1^ min^−1/2^)	*C*_1_	*R*_1_^2^	*k*_2_ (mg g^−1^ min^−1/2^)	*C*_2_	*R*_2_^2^	*k*_3_ (mg g^−1^ min^−1/2^)	*C*_3_	*R*_3_^2^
85.55	483.84	0.953	33.13	287.57	0.982	−9.95	129.75	0.431

Table 3 compares the adsorption capacities of cellulose-based and MOF-199-based adsorbents for MB.

**Table 3:** Comparison of adsorption capacity of MB by different cellulose-based adsorbents and MOF-199-based adsorbents

Absorbent	*q*_m_ (mg g^−1^)	Year
HKUST-1	454	2017 (ref. 37)
CS/CNF	164.5	2019 (ref. 42)
HKUST-1	5	2014 (ref. 38)
RCE/GO (0.5 wt%)	68	2018 (ref. 9)
HKUST-1@ABS	64.3	2014 (ref. 43)
HKUST-1/CCSA	526.3	2021 (ref. 20)
CCSA	254.1	2021 (ref. 20)
HKUST-1@Fe_3_O_4_	245	2020 (ref. 44)
MOF-199/CCF	659.6	This work

#### 3.3.6 Recycling performance of MOF-199/CCF

**Fig. 7:**
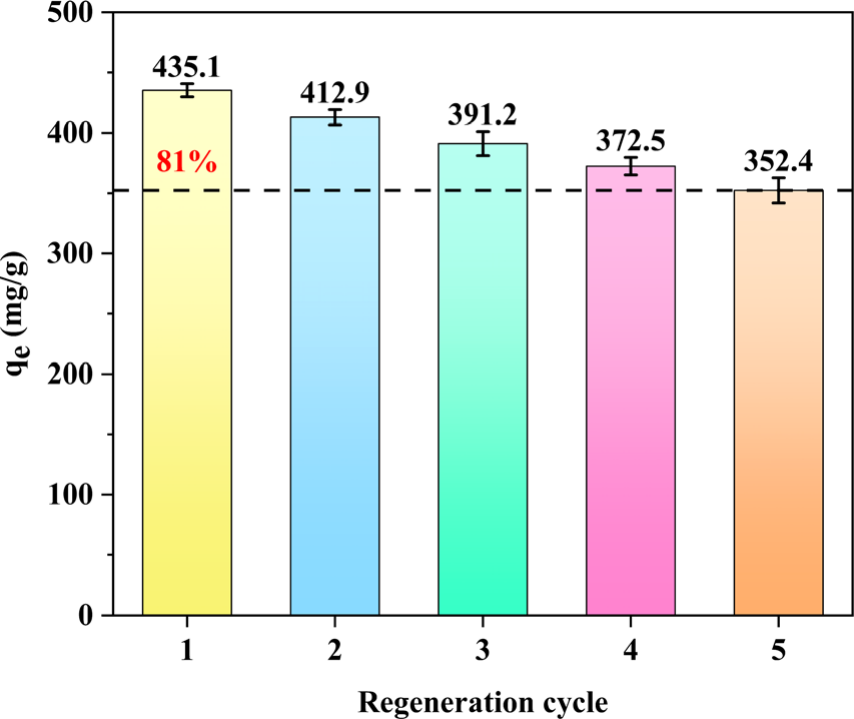
Cyclic adsorption performance of the MOF-199/CCF for MB adsorption.

The cyclic adsorption performance of the adsorbent is an important indicator for evaluating its quality. Fig. 7 shows the change in the adsorption capacity of the adsorbent on MB after 5 cycles. The adsorption capacity decreased from 435.1 mg g^−1^ to 352.4 mg g^−1^ after 5 cycles, but the equilibrium adsorption capacity can still reach more than 80% of the fresh adsorbent despite 5 cycles. MOF-199/CCF was subjected to a series of tests to assess its water resistance and cyclic stability. The SEM and PXRD characterisation of MOF-199/CCF after 5 days of immersion in deionised water and 5 cycles of cyclic adsorption, respectively, are presented in Fig. S8 and S9.† Fig. S8† illustrates the morphological changes that occur on the surface of MOF-199/CCF following water soaking and adsorption cycling. Initially, the particles aggregate into a particular morphology, but following this, a strip-like shape emerges, accompanied by the phenomenon of shedding. This phenomenon was more pronounced after five days of soaking in water compared to the cyclic adsorption of the phenomenon of shedding five times. This was due to the longer contact time between MOF-199/CCF and water compared to the cyclic adsorption of the phenomenon of adsorption of five times, which indicates that MOF-199 has poor water stability, it is a major reason for limiting its application, this also elucidates the diminished adsorption of MOF-199/CCF. As illustrated in Fig. S9,† the intensity of the characteristic peaks of MOF-199 was markedly diminished following immersion in water and adsorption cycles. However, the corresponding diffraction peaks remained evident, with the intensity of the characteristic peaks after cyclic adsorption being higher than that after water immersion. This suggests that the cyclic adsorption process resulted in a more robust crystalline structural integrity, in accordance with the SEM results. The intensity of the corresponding 22.5° diffraction peak of CCF is high in both cases, which is caused by the detachment of MOF-199 from the surface of CCF. These results indicate that MOF-199/CCF maintains the integrity of the crystal structure after five cycles. This proves that MOF-199/CCF has good reusability for the adsorption of MB. In conclusion, the results demonstrate the effectiveness of MOF-199/CCF as an adsorbent for MB.

## 4 Conclusion

In summary, we prepared a recyclable MOF-199/CCF sorbent with a high adsorption rate and large capacity for MB using a simple, safe, and rapid ultrasonic method at room temperature. The introduction of MOF-199 successfully improved the CCF and enhanced its adsorption performance for MB. CCF is an excellent carrier of MOF-199. The presence of CCF improves the agglomeration of small-sized MOF-199 prepared by ultrasonic method and greatly enhances the performance of MOF-199. The study analyzed the adsorption process of MOF-199/CCF and found that it is a combination of chemical and physical adsorption. The adsorption of MB by MOF-199/CCF is a clear example of monolayer adsorption. The process is exothermic and spontaneous, with a decrease in adsorption efficiency at higher temperatures. This work offers theoretical and technical support for utilizing ultrasonic methods in designing and preparing cellulose-based adsorbent materials. It also expands the application of cellulose-based adsorbent materials in the field of sewage treatment.

## Conflicts of interest

There are no conflicts to declare.

## Supplementary Material

RA-014-D4RA02099E-s001
