# Supplementary material for: Ultrasonic-assisted in situ synthesis of MOF-199 on the surface of carboxylated cellulose fibers for efficient adsorption of methylene blue
Source: RSC Adv. 2024 May 8;14(21):15095–105. doi: 10.1039/d4ra02099e (PMC11078215; doi:10.1039/d4ra02099e)
Supplement: RA-014-D4RA02099E-s001 [file RA-014-D4RA02099E-s001.pdf]

Electronic Supplementary Material (ESI) for RSC Advances.
This journal is © The Royal Society of Chemistry 2024

Supplementary information

Ultrasonic-assisted in-situ synthesis of MOF-199 on the surface of carboxylated cellulose fibers for efficient adsorption of methylene blue

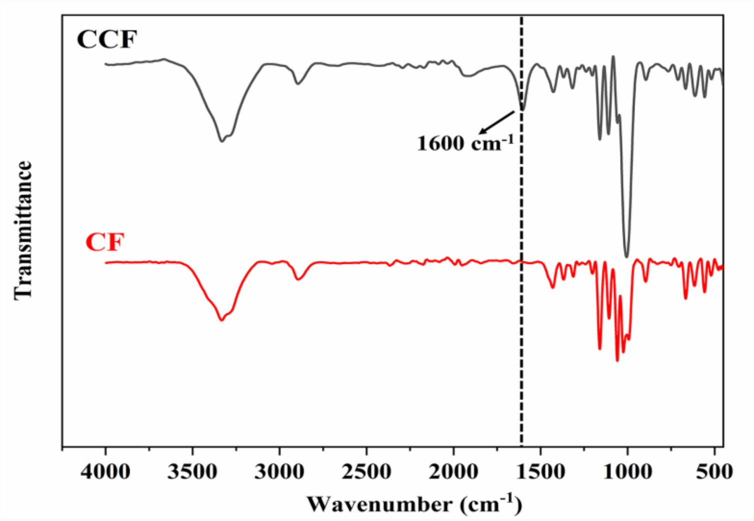

Fig. S1. FTIR spectra of CCF and CF.

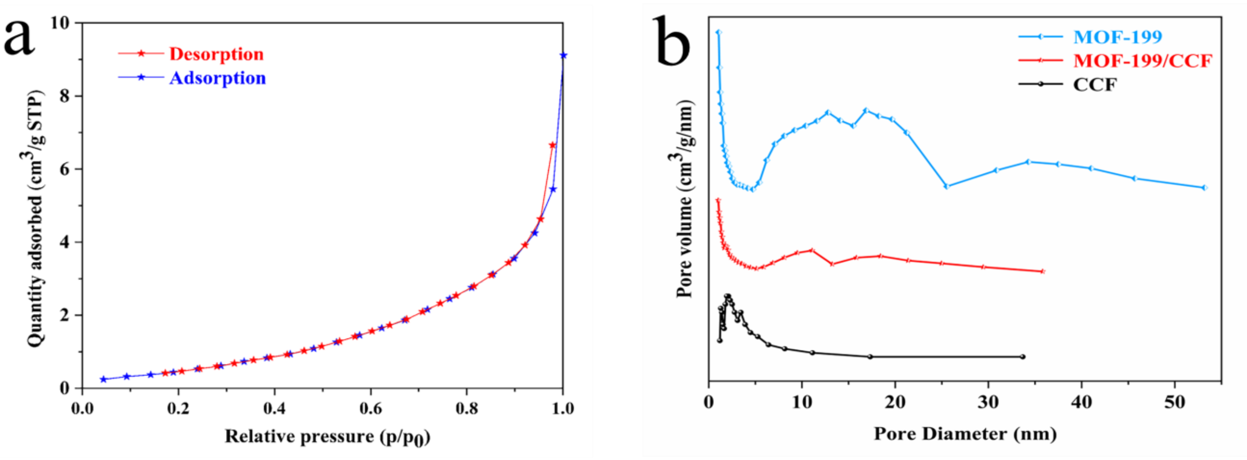

Fig. S2. N_2 adsorption-desorption curves of CCF (a); Pore diameter distribution of the three materials (b).

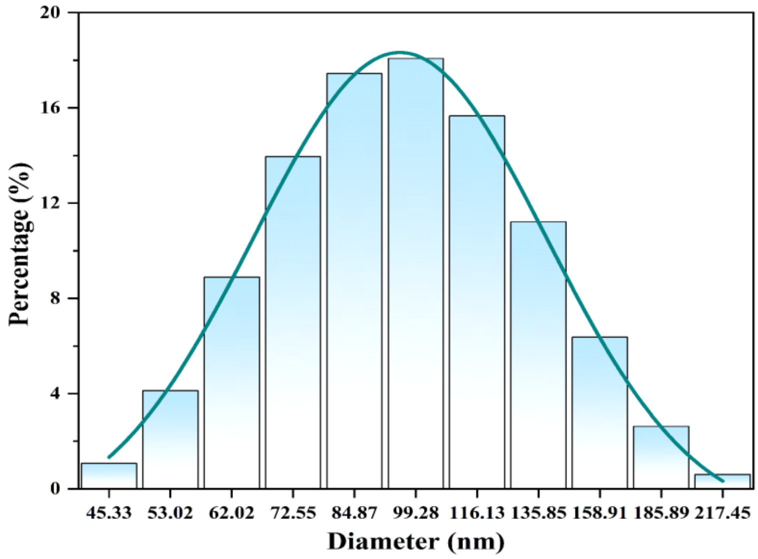

Fig. S3. Particle size distribution of MOF-199.

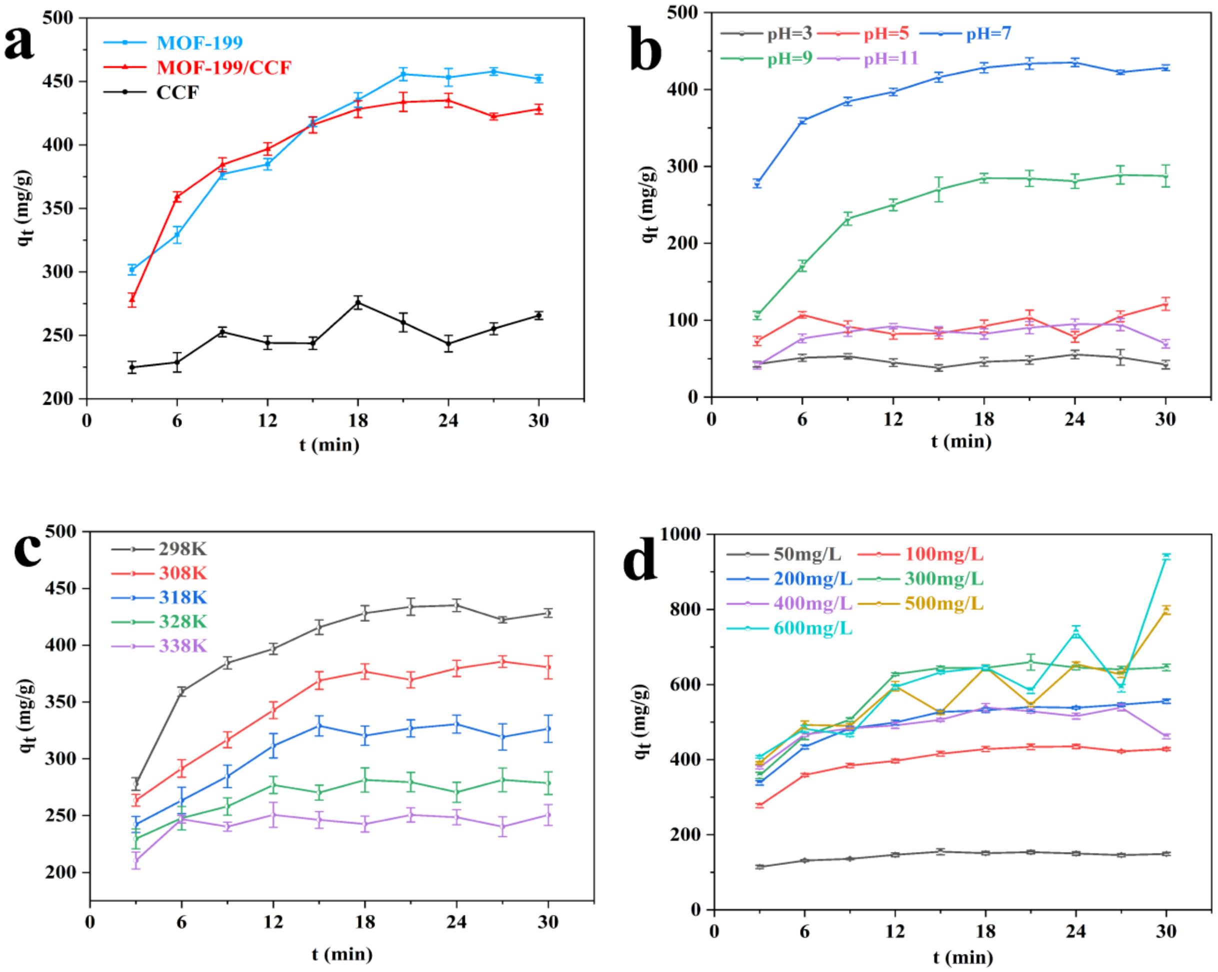

Fig. S4. Adsorption capacity over time: different adsorbents (a); pH (b); temperature (c); initial concentration (d).

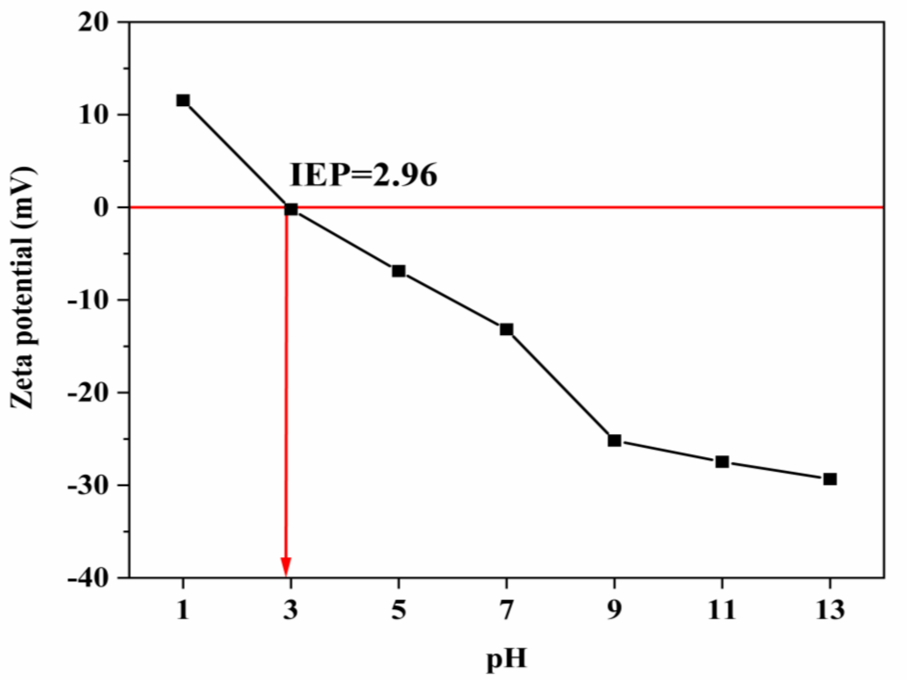

Fig. S5. The isoelectric point (IEP) of MOF-199/CCF.

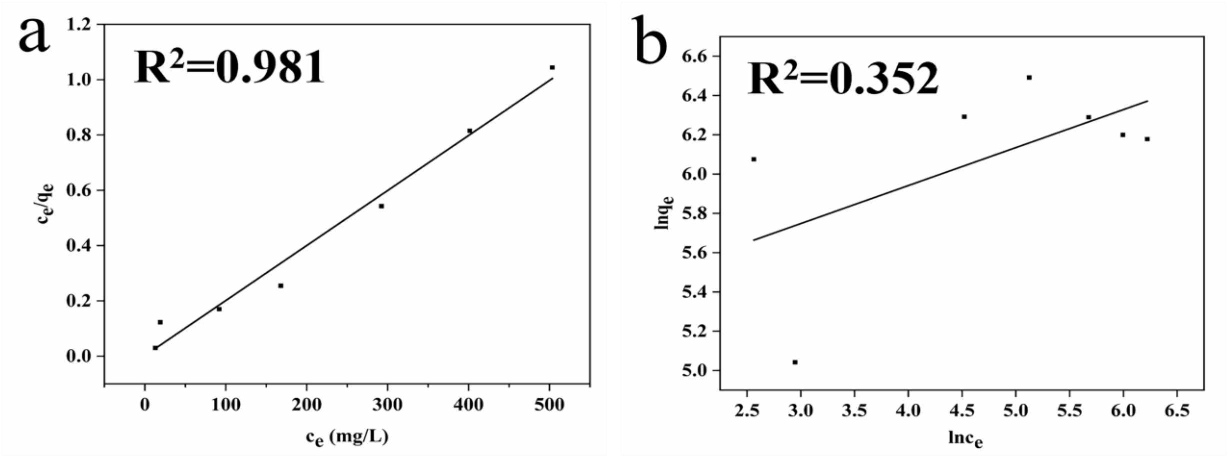

Fig. S6. Fitting results of MB on MOF-199/CCF by using Langmuir adsorption isotherm model (a); Fitting results of MB on MOF-199/CCF by using Freundlich adsorption isotherm model (b).

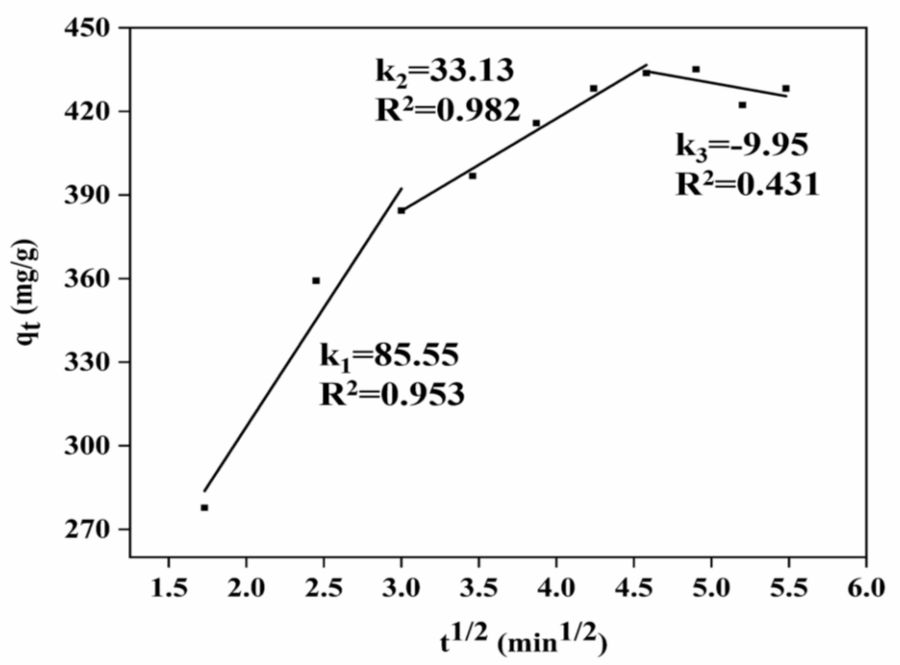

Fig. S7. Fitting results of MB on MOF-199/CCF by using Intra-particle diffusion model.

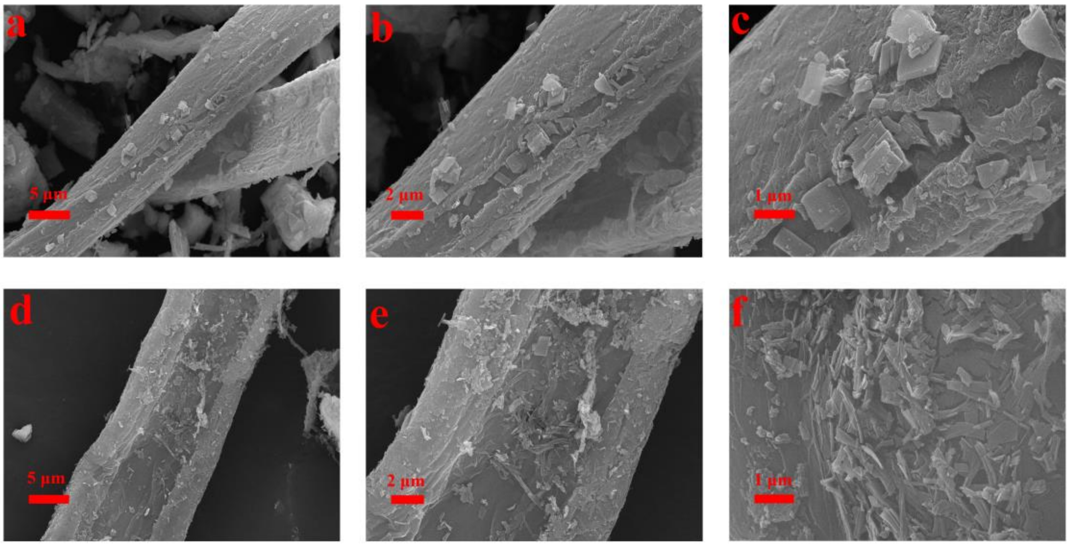

Fig. S8. After soaking in water for 5 days (a), (b) and (c); After 5 cycles of adsorption (d), (e) and (f).

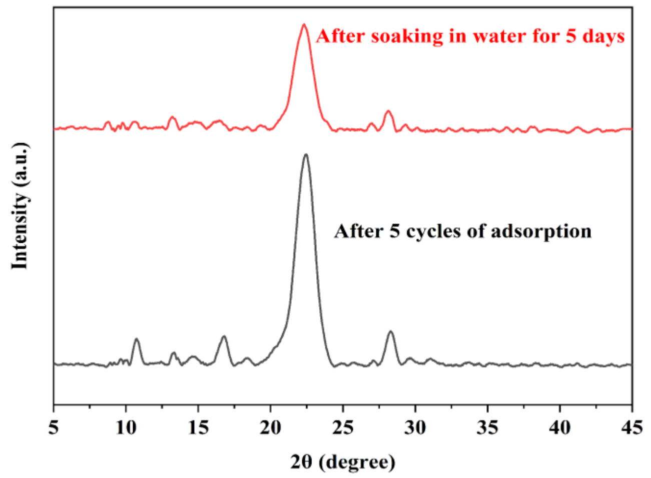

Fig. S9. PXRD characterisation of MOF-199/CCF.

Calculation method for percent composition of MOF-199/CCF.

The amounts of MOF-199 growth in the MOF-199/CCF was calculated by ash content. In order to calculate the loading of MOF-199 on CCF, the thermogravimetric behaviors of MOF-199, MOF-199/CCF and CCF from room temperature to 800 °C were characterized by TGA. As shown in Fig. 2(b), the ash generation ratio of MOF-199, MOF-199/CCF and CCF are 33.55 wt%, 15.54 wt% and 2.70 wt% respectively. If we assume that the mass of MOF-199 in 100g MOF-199/CCF is (x) g, then the mass of CCF is ($100\text{-}x$) g. And the mass loading ratio of MOF-199 on CCF can be calculated by Eq. S(1) and Eq. S(2):

$$x \cdot 33.55\% + (100 - x) \cdot 2.70\% = 100 \cdot 15.54\% \quad \text{S(1)}$$

$$W_{MOF\text{-}199} = x / 100 \cdot 100\% \quad \text{S(2)}$$

where $W_{MOF\text{-}199}$ represents the mass loading ratio of MOF-199 on CCF. Combined with Eq. S(1) and Eq. S(2), it is calculated that the mass loading ratio of MOF-199 on CCF is around 41.62 wt%.

Table S1

BET specific surface area and average pore diameter of the three materials.

MOF-199		MOF-199/CCF		CCF	
BET specific surface area (m^2/g)	Average pore diameter (nm)	BET specific surface area (m^2/g)	Average pore diameter (nm)	BET specific surface area (m^2/g)	Average pore diameter (nm)
645.26	2.19	264.83	2.53	2.31	12.3

Table S2

Adsorption thermodynamic model parameters of MOF-199/CCF adsorption of MB.

ΔH ($KJ·mol^{-1}$)	ΔS ($J·K^{-1}·mol^{-1}$)	ΔG ($KJ·mol^{-1}$)				
		298K	308K	318K	328K	338K
-40.09	-106.50	-8.35	-7.29	-6.22	-5.16	-4.09

Table S3

Pseudo-first-order kinetic and pseudo-second-order kinetic model parameters for the adsorption of MB by MOF-199/CCF.

q_e (mg/g) experimental	Pseudo-first-order model			Pseudo-second-order model		
	q_e (mg/g) theoretical	k_1 (min^{-1})	R^2	q_e (mg/g) theoretical	$k_2 \times 10^{-3}$ ($g·mg^{-1}·min^{-1}$)	R^2
435.1	418.1	0.2397	0.922	454.5	1.35	0.998
